# Constructing an Interactive and Integrated Analysis and Identification Platform for Pathogenic Microorganisms to Support Surveillance Capacity

**DOI:** 10.3390/genes14122156

**Published:** 2023-11-29

**Authors:** Yang Song, Songchao Zhong, Yixiao Li, Mengnan Jiang, Qiang Wei

**Affiliations:** 1 National Key Laboratory of Intelligent Tracking and Forecasting for Infectious Diseases, Chinese Center for Disease Control and Prevention, Beijing 102206, China; songyang@chinacdc.cn; 2 National Pathogen Resource Center, Chinese Center for Disease Control and Prevention, Beijing 102206, China; zhongsongchao@126.com (S.Z.); liyix2@chinacdc.cn (Y.L.); jiangmn@chinacdc.cn (M.J.)

**Keywords:** platform, pathogenic microorganisms, identification

## Abstract

Introduction: Whole genome sequencing (WGS) holds significant promise for epidemiological inquiries, as it enables the identification and tracking of pathogenic origins and dissemination through comprehensive genome analysis. This method is widely preferred for investigating outbreaks and monitoring pathogen activity. However, the effective utilization of microbiome sequencing data remains a challenge for clinical and public health experts. Through the National Pathogen Resource Center, we have constructed a dynamic and interactive online analysis platform to facilitate the in-depth analysis and use of pathogen genomic data, by public health and associated professionals, to support infectious disease surveillance framework building and capacity warnings. Method: The platform was implemented using the Java programming language, and the front-end pages were developed using the VUE framework, following the MVC (Model–View–Controller) pattern to enable interactive service functionalities for front-end data collection and back-end data computation. Cloud computing services were employed to integrate biological information analysis tools for conducting fundamental analysis on sequencing data. Result: The platform achieved the goal of non-programming analysis, providing an interactive visual interface that allows users to visually obtain results by setting parameters in web pages. Moreover, the platform allows users to export results in various formats to further support their research. Discussion: We have established a dynamic and interactive online platform for bioinformatics analysis. By encapsulating the complex background experiments and analysis processes in a cloud-based service platform, the complex background experiments and analysis processes are presented to the end-user in a simple and interactive manner. It facilitates real-time data mining and analysis by allowing users to independently select parameters and generate analysis results at the click of a button, based on their needs, without the need for a programming foundation.

## 1. Introduction

Infectious diseases caused by pathogenic microorganisms such as bacteria and viruses are one of the main causes of human death and disease worldwide [1,2]. Early identification of infected patients and timely identification of pathogens and antimicrobial resistance characteristics can help improve clinical treatment effectiveness and reduce adverse outcomes [3,4]. With the rapid development of high-throughput “next-generation sequencing (NGS)”, WGS analysis has become a powerful tool for the detection, identification, and analysis of human pathogenic microorganisms. Advances in this method have made it possible to quickly obtain detailed sequence data required for pathogen identification and analysis. Compared with traditional methods, the genome sequences generated using NGS technology could be rapidly and accurately utilized to detect and identify pathogens, assess pathogenic factors such as antibiotic resistance and virulence, obtain pathogenic information as early as possible, and support diagnostics [5,6,7]. In addition, numerous studies have demonstrated the enormous potential of WGS in epidemiological investigations, including the revelation of the origin and transmission pathway of pathogens through WGS, becoming the preferred method for outbreak investigation and pathogen monitoring [5,8]. Also, the World Health Organization (WHO) and their partners are launching a global pathogen monitoring network, based on pathogenic sequencing, to help protect people from the threat of infectious diseases with the power of pathogen genomics [9].

Although WGS technology plays an important role in clinical, scientific, and public health fields, how to utilize these massive sequences and obtain key information from them has become a challenging task in biological research. Firstly, the splicing, annotating, and analysis of sequencing data requires the use of large amounts of bioinformatics software, which poses a daunting challenge to biomedical researchers without programming experience. Moreover, the hardware costs of data analysis have increased significantly, due to the large amount of data generated via high-throughput sequencing, which also escalated the computational requirements for data throughput and efficiency [10]. The genome sequences of pathogenic microorganisms are becoming increasingly available as genome sequencing technology improves and sequencing costs continue to decrease. For pathogenic microorganisms in particular, there is an urgent need for an integrated database and platform with analytical capabilities to continuously integrate, collect, and manage different types of data so that these resources can be efficiently used for the analytical identification of pathogenic microorganisms, subsequent research applications, and to improve public health decision making.

In order to improve the analysis and utilization capabilities of pathogen genomes and deepen the development of pathogen genome data resources, taking *Rhodococcus equi* as an example, we should combine the relevant data information of *R.equi*, integrate strain resources and database resources with the analysis platform, and effectively organize the information to provide a dynamic interactive online analysis platform (http://123.57.82.208:8082/#/rhodococcus/homepage, accessed on 28 November 2023) for user exploration. The platform will present the complex background experiment and analysis process to the end-user in a simple and interactive way, through the encapsulated design of the cloud service platform, which is applicable to the deep genomic analysis of all pathogens. Such a platform will enable research, and medical and public health professionals without a bioinformatics foundation to independently select parameters and generate relative analysis results with a single click, and to have fully dynamic graphics according to their needs, facilitating real-time data mining and analysis. Simultaneously, the problem of personal computers and workstations unable to complete data processing and the low efficiency of existing manual business processes can be solved using cloud computing platforms. The planned system will provide improvements in levels of diagnosis, prevention, and control of pathogenic bacteria, while promoting the exploration and use of information resources on pathogenic microorganisms.

## 2. Methods

### 2.1. Platform Design

**Figure 1:**
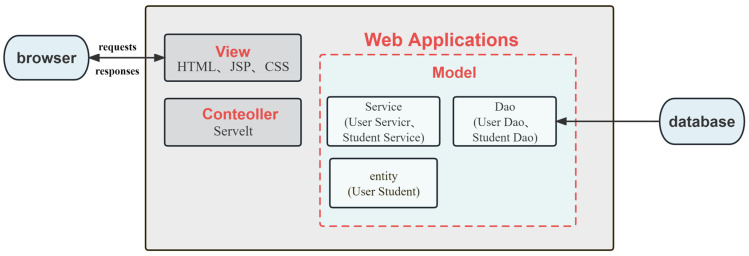
Interactive system (MVC mode).

The application was constructed using the Java programming language. The user interface utilizes the VUE framework along with the MVC (Model–View–Controller) pattern to enable interactive service functions for both front-end data collection and back-end data calculation (Figure 1). The Model constitutes the primary component of the application program, incorporating object entity class beans for storing business data, and business-processing beans for processing business logic and handling database access. The View is a resource used in applications to interact with web browsers and exhibit information. The resource possesses a specific use case for facilitating user engagement via a graphical user interface. Technical abbreviations will be defined upon their introduction, and value-neutral language will be used throughout to avoid emotional rhetoric. The structure will follow accepted academic guidelines, with an emphasis on causal connections and logical flow of information. All grammar and spelling will conform to British English standards. In this platform application, the View comprises a front-end page that utilizes HTML, CSS, JavaScript, and other technologies to interact with browsers and exhibit data resources. The Controller, on the other hand, refers to the Servlet component of an application program, which relays user requests to the model layer for processing, and subsequently returns the processed data to the View for rendering and user interaction. Sensible structuring ensures the logical flow of information with causal connections between statements, while precise language and technical vocabulary help convey the meaning more accurately. Spelling, grammar, and style align with British English conventions. The system utilizes a MySQL database, operates on a Linux operating system, and follows a Browser/Server structure with Multitier architecture.

### 2.2. Business Model

**Figure 2:**
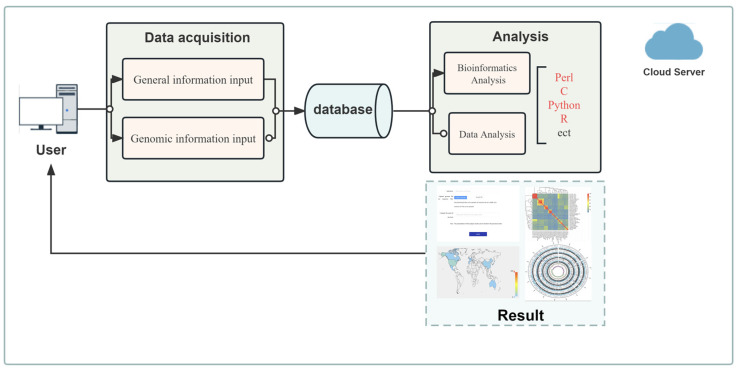
Integrated analysis model of gene data.

For the genetic data analysis application, we have devised a business model which integrates cloud computing services with map components, bioinformatics analysis tools, and scripts in Perl, C, Python, and R computer languages. See Figure 2 for details. This model carries out sequencing data analysis through the dynamic interactive analysis module, achieving data collection upon receipt of task execution commands. The dynamic interaction analysis module utilizes cloud computing storage to analyze, calculate, and store data.

## 3. Results

### 3.1. Database Content

#### 3.1.1. Overview

This platform has been designed and constructed to offer a novel visual interaction system, capable of performing data upload, download, and analysis, without requiring the end-user to possess cumbersome programming code or parameter setting knowledge. The intuitive visual interface, built using JavaScript, allows users to set parameters through mouse clicks and input, thus allowing for the easy attainment of results, thus achieving our goal of “WYSIWYG (What You See Is What You Get)”. Furthermore, the platform enables users to export their research outcomes in varying formats, beyond the scope offered.

**Figure 3:**
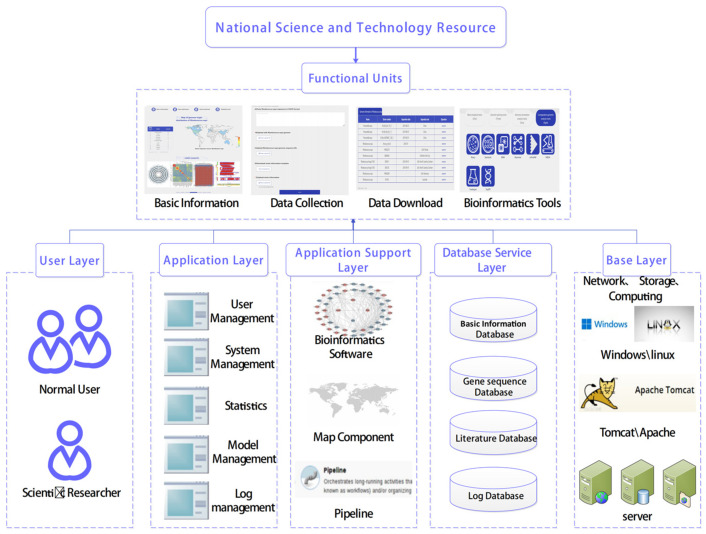
Platform overview. The platform consists of four main functional units: (1) basic information; (2) data acquisition module; (3) data download module; (4) online analysis tool module. The user layer, service application layer, data download layer, data layer, and basic layer provide technical support for each functional module.

The platform assembled consists of four modules: basic information, data collection, data download, and online analysis tools. This indispensable NPRC unit can be accessed by users through the official website homepage by logging on via (https://www.nprc.org.cn/#/rhodococcus/homePage, accessed on 28 November 2023) or searching for “NPRC”. Users can register their account information on the platform, perform login operations, and obtain login information. Based on the login information, the platform determines the permissions of the login user. It subsequently provides an interface for the login user to use the cloud platform for pathogen genome sequencing for biological information analysis, and quickly relays the results to the user’s account after the user performs the operation (Figure 3).

#### 3.1.2. Basic Information

To address the requirements of biomedical researchers, the database has been categorized into two sections: basic and characteristic databanks. Technical terms have been clarified to facilitate comprehension. Consistent citation and formatting rules have been applied while avoiding filler words, grammatical errors, and punctuation errors. The basic section comprises a gene sequence database, basic information database, literature database, and log database, which allow for generic research on pathogenic microorganisms. Causal connections are established to ensure a logical flow of information. Language is objective and value-neutral, while maintaining a formal register. The database features a resource database for equine streptococcus, a disease flow database, and a bioinformatics database. Its primary utility lies in the detection and identification of various bacteria, including equine streptococcus. Data are separated into international and domestic categories, with primary sources such as NCBI contributing towards the international aspect. The domestic data comprise collections from clinical experiments, projects, and other such sources.

#### 3.1.3. Data Management

##### Data Sources

At present, the platform has archived the genomes of 83 *Rhodococcus equi* strains, all from the public database (NCBI). The genome is a publicly available sequence submitted by various institutions around the world, with four strains from the NPRC. The platform has built a total of 13 bioinformatics analysis tools, with software source code available from the open-source code hosting platform Git Hub (https://github.com, accessed on 28 November 2023).

##### Uploading, Downloading, and Sharing of Public Data

The platform includes a system for user registration and account information, facilitating subsequent login and operations. The login information is used to determine the login user’s permissions. An interface is provided for login users to access the cloud platform for pathogen genome sequencing and biological information analysis. The user’s genome and analysis result files are securely stored in the analysis directory, accessible only through their account. Technical terms are explained when first used. The structure and language adhere to academic writing principles, and relevant style guides are followed. Grammatical correctness and balanced language are ensured throughout. We encourage users to submit new genome sequences and strains. However, it is necessary to obtain permission to retain any genomic data. The following functional operations are specified:

1. Task Viewing Unit

Users are able to access the results obtained from the analysis tool within this unit. These results comprise numerous files in varying formats, including fna, ffn, and gff formats, as well as a selection of charts and image files, which users have the option to save for future use.

2. Data Upload Information Viewing Unit

Users can access genomic data records uploaded by individuals in the background. Technical abbreviations will be defined upon first use. The data module enables users to upload their own data, view data that has been uploaded, and monitor the upload progress in real time. Common academic sections and formatting conventions will be used throughout to maintain consistency. The data source consists of pathogenic bacteria de novo sequencing data obtained from various platforms, as well as the annotation file obtained from the biological information database. The data format can be any one of .fna, .gz, .faa, .fastq, .gff2, .gff3, or .vcf. In order to improve the efficiency of resource utilization, this platform offers a method of generating a dynamic and interactive cloud based microbiome analysis platform. The dynamic interactive analysis module provides chart tools that can modify color schemes, shape schemes, and column directions, as well as present legends, point names, and merge or sort functions. The findings from the dynamic interaction analysis module can be saved in a report and presented within the same report. Additionally, the analysis graph can be obtained in PNG, JPG, PDF, and SVG formats.

#### 3.1.4. Online Bioinformatics Analysis Tool

The platform offers a range of analytical tools. These tools involve database construction, remote visual calling, and bioinformatics analysis of genomics data. Furthermore, the distributed architecture of the platform’s front- and rear-end separation is used. The front end of the system utilizes the Vue framework and html, as well as jQuery, Echarts, and other technologies, to facilitate visual interaction with users. Users may choose relevant analysis tools based on their requirements and enter the appropriate genome data. Intensive data computation employs encapsulated bioinformatics analysis code scripts. Once different parameters have been selected and submission completed, the back end of the system handles and renders data. After processing is finished, the back end transmits the outcomes to the front end, which enables users to obtain the analysis results. It is vital to explain the technical term acronyms when first introduced.

##### Bioinformatics Analysis Tools

**Figure 4:**
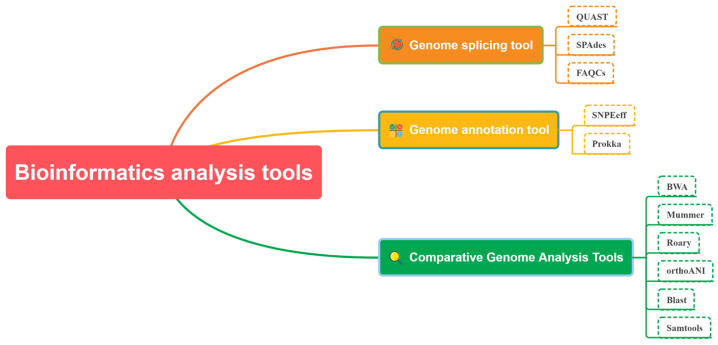
Bioinformatics Analysis Tools.

The platform provides several analysis tools that are categorized according to various sequencing methods. These tools can be classified into four main types based on their functions, namely comparative genome analysis, genome splicing, genome annotation analysis, and Blast analysis tools. Examples of such tools comprise Samtools, BWA, QUAST, SPAdes, Prokka, SnpEff, Mummer, Roary, Freebayes, Blast, orthoANI, FAQCS, and others (Table 1). The sequence assembly tools assess gene sequence quality and effectively construct gene outlines from short-read long or long-read long fragments. Blast tool construction is facilitated by sequence alignment, where users select appropriate parameters to determine alignment requirements for two sequence fragments. Genome annotation encompasses gene annotation, translation, and other relevant factors; the resulting files may be used for further analysis. Comparative genomic analysis involves various essential tools for conducting research, including pan-genomic analysis tools. These tools can meet basic bioinformatic analytical requirements for bacterial pathogens (Figure 4).

##### Online Interaction Analysis

After selecting the relevant analysis tool, the user uploads the genome information and enters the file name. The user then sets the parameters and submits the data analysis according to the process prompted on the webpage. Finally, the Taskbar in the personal center displays the analysis results.

### 3.2. Case Study 1

**Figure 5:**
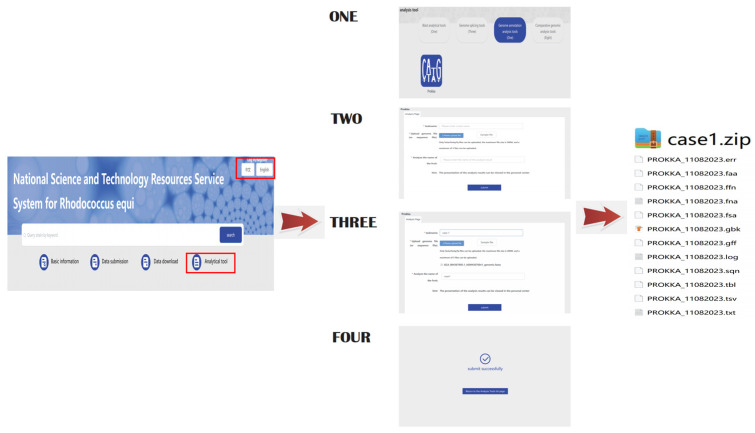
Genome annotation demonstration process.

This case study employed a genome of *R.equi* to simulate the process of genome annotation. The software tool developed on this platform is a versatile bioinformatics tool that can be used for analyzing genomes of a range of bacteria, not just *R.equi*. First-time users are required to create an account, as it is the only way to manage result data. Furthermore, since our platform is part of the NPRC, this account can potentially enable strain submission and other similar functions. Users can navigate to the analysis tool module on the platform page, proceed to click on the annotation tool ‘prokka’, and follow the process prompts to upload the genome file to initiate the analysis operation. Once the analysis process is finished, users can access the results via their account (Figure 5).

## 4. Discussion

The pathogenetic genome holds the genetic code of viruses, bacteria, and other pathogenic microorganisms. This assists scientists and public health officials in comprehending their infectivity, pathogenicity, and means of transmission, in turn enabling the detection and monitoring of diseases, thus obviating and responding to epidemics. NGS technology has demonstrated its ability to effectively trace the origin, transmission, and progression of epidemics, as well as monitor the development of pathogens. The response to emerging infectious diseases like SARS, MERS, Zika, and Ebola has highlighted the efficacy of NGS technology in these areas [11,12,13]. The COVID-19 outbreak has also emphasized the significance of pathogen genomics in handling pandemics. For instance, the use of NGS technology and computational tools enabled the identification of SARS-CoV-2 within a few days of the reporting of novel coronavirus pneumonia. Genome sequencing-based molecular epidemiology assisted in the investigation of the emergence, transmission, and tracking of SARS-CoV-2 during the pandemic [14]. On 20 May 2023, the WHO announced the launch of the International Pathogen Monitoring Network. The new network will rely on pathogen genomics to conduct genetic analyses of viruses, bacteria, and other pathogens. The aim is to understand the infectivity, case fatality, and mode of transmission of disease. It hopes to use pathogen genomics to help protect people from the threat posed by infectious disease [9]. Setting up a local genomic surveillance program requires appropriate infrastructure, including patient-focused diagnostic laboratories to identify and acquire samples containing target pathogens, MRC molecular biology laboratories, advanced sequencing infrastructure, and bioinformaticians to process and analyze data [15]. The accessibility of these resources differs across regions. Despite COVID-19 having enhanced genomic capacity in countries recently, numerous nations still lack efficient systems for sampling and analyzing data or utilizing this information for public health decisions.

Using *R.equi* as an illustration, this platform integrates the NPRC’s strain and genome resources with relevant data on *R.equi* to offer a dynamic and interactive online tool for biological information analysis. There are presently numerous network servers that offer bioinformatic analysis services within a pipeline composition [16,17,18]. Our platform is focused on treating pathogenic bacterial strains and their genomes as a cohesive unit. Our approach provides users with comprehensive samples, data, and analysis tools, as well as independent tools, with the aim of creating a flexible and adaptable analysis mode. This mode can be combined by users in a manner that best suits their needs, ultimately reducing unnecessary processes and saving time. The platform offers services while also collecting strains and data, with the objective of becoming a comprehensive biological sample platform for infectious disease research that benefits public welfare. Our aim is to enhance international projects for pathogen surveillance networks and promote better decision making in public health. This will be achieved by enhancing the capacity of China, and other countries, to utilize microbial resources and genomic surveillance of pathogenic agents. By fully integrating pathogen genome information with clinical and public health systems, we strive to attain this goal. Currently, the platform is solely capable of facilitating bacterial genome analysis. Nevertheless, its capability is restrained due to insufficient server capacity and processing power, limiting its ability to render analytical solutions only to selected Chinese users. In the future, the platform’s capability to analyze and identify an increased variety and amount of pathogenic microorganisms will be enhanced. Furthermore, the server capacity will be expanded to allow for analysis and identification services for a vast array of pathogens, encompassing bacteria, viruses, and fungi, to be provided to a greater number of users. The platform shall integrate artificial intelligence algorithms and methods to train models for prompt and precise identification of pathogens, facilitating early monitoring and warning of infectious diseases.

## Figures and Tables

**Table 1:** Tools embedded in the platform.

Type	Tool	Reference Websites *
Blast analytical tools	Blast	https://blast.ncbi.nlm.nih.gov/doc/blast-help/downloadblastdata.html
Genome splicing tools	QUAST	https://github.com/ablab/quast
	SPAdes	https://github.com/ablab/spades
	FaQCs	https://github.com/LANL-Bioinformatics/FaQCs
Annotation analysis	Prokka	https://github.com/tseemann/prokka
comparative genome analysis	Roary	http://sanger-pathogens.github.io/Roary/
	Samtools	https://github.com/samtools/samtools
	BWA	https://github.com/lh3/bwa
	Mummer	https://github.com/mummer4/mummer
	othoANI	http://www.ezbiocloud.net/sw/oat
	MEGA	https://www.megasoftware.net/
	Freebays	https://github.com/ekg/freebayes
	SnpEff	https://pcingola.github.io/SnpEff/

* Websites accessed on 28 November 2023.

## Data Availability

No new data were created or analyzed in this study. Data are contained within the article.
